# Electroconvulsive Therapy for a 14-Year-Old Patient With Autism and Refractory Agitation: A Case Report

**DOI:** 10.1192/bjo.2025.10746

**Published:** 2025-06-20

**Authors:** Fouad Sabatin, Nizar Marzouqa, Rasmea Asad, Aya Alshalash, Batoul Housheya

**Affiliations:** 1 Hebron University, Hebron, Palestine; 2 Bethlehem Psychiatric Hospital, Bethlehem, Palestine

## Abstract

**Aims:** To present a case of refractory agitation in a patient with autism spectrum disorder (ASD) and explore electroconvulsive therapy (ECT) as a therapeutic option.

Supervision was provided by Dr Iyad Alazzeh (Halhul Community Mental Health Center).

**Methods:** A 14-year-old male patient with ASD presented to his psychiatrist after several failed attempts to integrate him into special-care schools due to increasing disturbance of behaviour. He has no other medical problems, and he lives with his father, who is diagnosed with schizophrenia, and his grandmother. His mother often visits him and helps take him to medical appointments. Several pharmacological agents have been attempted: Valproic acid, clonazepam, risperidone, olanzapine, and chlorpromazine, without improvement in his condition. The child’s condition further deteriorated as he stopped accepting medications, which disrupted his sleep and caused bursts of laughter and screaming. During an appointment, the patient attacked his mother and bit her causing an injury necessitating medical intervention. After a multidisciplinary evaluation, obtaining informed consent, and familiarizing the patient with the setting, electroconvulsive therapy (ECT) was initiated as a last resort. The patient underwent a series of eight ECT sessions under general anaesthesia.

**Results:** The only documented side effect was irritability at bedtime on the day of each ECT session, which disappeared with sleep. After he finished all his sessions, the patient had decreased laughter, started accepting medications again, was more responsive to directions, and didn’t exhibit aggression. However, his baseline agitation didn’t significantly improve, leading to the persistence of social integration challenges. It is difficult to determine if the cessation of physical aggression was the result of ECT or resuming medications.

Research on ECT use in paediatric populations is limited but growing, with studies indicating its potential to address severe neuropsychiatric symptoms, including catatonia, mood dysregulation, and treatment-resistant aggression. ECT has shown efficacy in managing specific refractory symptoms, particularly in cases where pharmacotherapy and behavioural interventions fail. However, ethical concerns, stigma, and limited clinical trials have historically restricted its use in this population.

**Conclusion:** Severe agitation in paediatric patients with autism spectrum disorder (ASD) presents a significant therapeutic and diagnostic challenge. This case highlights the potential of ECT to target specific refractory behaviours in paediatric patients with neuropsychiatric conditions. Further research into the role of ECT in managing treatment-resistant agitation in children with ASD is required.

